# Rare Disease Education Outside of the Classroom and Clinic: Evaluation of the RARE Compassion Program for Undergraduate Medical Students

**DOI:** 10.3390/genes13101707

**Published:** 2022-09-23

**Authors:** Ari Morgenthau, Colton Margus, Michael P. Mackley, Ashley P. Miller

**Affiliations:** 1 Department of Medicine, Division of Endocrinology and Metabolism, University of Toronto, Toronto, ON M5S 3H2, Canada; 2 Department of Emergency Medicine, Bronxcare Health System, The Bronx, NY 10457, USA; 3 Department of Pediatrics, Division of Clinical and Metabolic Genetics, The Hospital for Sick Children, University of Toronto, Toronto, ON M5G 1X8, Canada; 4 Department of Medicine, Division of General Internal Medicine, Dalhousie University, Halifax, NS B3H 2Y9, Canada

**Keywords:** medical education, rare diseases, program evaluation, RARE Compassion Program, David R. Cox Scholarship

## Abstract

Launched in 2014, the RARE Compassion Program is the first international educational program to pair medical students with rare disease patients in order to enhance exposure to and comfort with rare diseases. As part of ongoing quality improvement, this study retrospectively reviewed four years of participant registration data to conduct a program evaluation of the RARE Compassion Program between 2014–2018. During the study period, there were 334 student participants, representing 67.3% of Association of American Medical Colleges (AAMC) member medical schools, and 5389 rare disease volunteers. Despite not requiring in-person interaction, 90.64% of student–volunteer interactions were in-person, while only 5.89% and 3.46% were by video messaging or email correspondence, respectively (*p* = 0.0002). In a limited post participation survey, 91.7% of students, who matched to 19 out of 27 residency specialities, indicated they would recommend the program to their peers. These findings suggest that the RARE Compassion Program, designed to increase medical student engagement with rare disease patients, has broad appeal. It serves as a novel case study of how extracurricular initiatives supported by non-profit organizations can augment the medical training experience and improve understanding of important and often neglected perspectives.

## 1. Background

There are nearly 10,000 unique known rare diseases, defined as having an incidence of less than 1 in 1500–2500 individuals [1,2,3,4]. These diseases vary widely in clinical presentation even among patients sharing the same diagnosis. While each rare disease only affects a small number of individuals, collectively they affect 1 in 11 Americans and 1 in 12 Canadians [5,6,7]. This is similar to the prevalence of many common chronic conditions, such as diabetes [8].

Unlike common chronic diseases, however, medical expertise in rare disease is uncommon, and the care offered is limited [1,5,6,9,10,11,12,13]. While common diseases receive ample focus in undergraduate medical education, there is less focus on rare disorders. The need for education focused on the recognition, understanding, and management of rare patients, particularly during medical school, was highlighted by the Association of American Medical Colleges (AAMC) in 2004 [14,15]. Since then, there has been an increased focus on integrating genetics and rare disease in undergraduate medical education, with the vast majority occurring in the preclinical years [15,16]. However, with 80% of rare disorders being genetic in origin, it is notable that only 11% of medical schools surveyed by Thurston et al. offered practical training in the clinical applications of medical genetics [15,17,18]. More recently, despite these changes, Haspel et al. and the Canadian Rare Disease Working Group report highlighted the ongoing need for further inclusion of rare disease education within undergraduate medical education [6,19].

The RARE Compassion Program is an educational experience aimed at meeting this need. It is a voluntary program, available to medical students, that pairs students with members of the rare disease community. Over several months, students are expected to interact with rare disease community members in order to develop a greater understanding of the unique rare disease patient experience. The RARE Compassion Program is an experiential learning program, intended to complement existing didactic learning in existing medical curricula. The aim of this study was to evaluate the impact of this novel program using a modified Kirkpatrick model. This program evaluation therefore explored participation and evaluated the responsiveness of students to a program focused on patients with rare diseases.

## 2. Methods

### 2.1. Context

The RARE Compassion Program is a voluntary, experiential learning program available to all undergraduate medical students. Originally named The David R. Cox Scholarship for Rare Compassion it was founded in 2014 as a collaboration between a small group of medical students and Global Genes, a third-party global rare disease patient and family advocacy organization. In 2019 the effort was expanded into the RARE Compassion Program, which matches healthcare trainees, including allied health fields, with patients and families affected by rare diseases (“rare volunteers”). In the earliest iterations, students were only matched with a single rare volunteer and their family. The program has since been updated to facilitate matching with multiple rare volunteers over the academic year. At the time of publication, participants can access and sign up for the program via its website https://globalgenes.org/compassion/, Last accessed on 28 August 2022. Once registered, students are matched with four rare volunteers (two months each), over an eight-month period. During each two-month cycle, students are encouraged to connect with their rare volunteer at least three times for 45–60 min. The underlying goal of the program was to provide future healthcare professionals with a greater understanding of these important illnesses, with a particular focus on the patient experience and diagnostic odyssey encountered by rare disease patients. Throughout the several months of their partnership, students and rare volunteers were free to communicate virtually or in-person. Where possible, student participants were matched by Global Genes with local rare volunteers. This was done to maximize the potential for in-person interactions, in the hope that they would develop a richer connection with their matched rare volunteers. At the end of the program, medical students specifically are asked to submit a reflective essay of their experience to be considered for the David R. Cox Scholarship, which comes with a monetary award. These essays were reviewed by a panel of reviewers, including rare disease patients, experts, and advocates, to select a recipient for a monetary award.

The program is communicated to students by Global Genes through multiple methods, including: emails to medical school administration, faculty, and student interest groups; social media outreach; rare disease blog posts; and, word of mouth. Similarly, rare volunteers are recruited by Global Genes using multiple avenues, including: individual rare disease advocacy groups; partner organizations; online blogs and articles; social media; and, word of mouth.

### 2.2. Participants and Data Collection

All data were collected as part of ongoing quality improvement and were thus exempt from Research Ethics Board review. Registration information for student participants from 2014 to 2018 was used for data analysis. Given that United Kingdom participants were only included through a soft launch late in 2018, students included in this study were limited to those attending AAMC member medical schools within the United States and Canada. Students without complete registration information were excluded from analysis. The following variables were extracted from the registration data and de-identified: participant school of study, type of match (individual patient or family), type of rare volunteer (adult or pediatric), and type of interaction with rare volunteer (in person, video messaging, or email). Field of practice was obtained for participants who had reported graduating prior to this evaluation using publicly available databases of medical licenses and residency program websites and placed in a separate de-identified dataset. As a measure of program competitiveness, the number of residency seats per United States applicant from the 2018 National Resident Matching Program (NRMP) was used for comparison, where applicable. Numbers of deidentified rare volunteers, with self-identification as pediatric versus adult, were provided by Global Genes.

### 2.3. Post Participation Survey

A brief post participation survey was sent to 53 students following completion of the 2018 program. Students were asked to rate the program using a Likert scale. Students were also asked a series of yes/no questions. First, would they recommend or have recommended the program to their peers? Second, do they plan to advocate for rare disease education within their medical school? Finally, do they plan to become involved with rare disease advocacy after medical school? Descriptive statistics were used to summarize these data.

### 2.4. Data Analysis

Given the difficulty in determining the number of students who had knowledge of the RARE Compassion Program, program uptake was analyzed relative to individual Liaison Committee on Medical Education (LCME) accredited medical schools rather than individual students. The analysis further sub-grouped schools by the presence of postgraduate medical education in Medical Genetics, which was considered as a marker of a center with affiliated rare disease infrastructure. A Chi-squared test of independence was calculated to compare observed and expected distribution of student participants among rare disease affiliated schools. Student residency fields were compared to the total available postgraduate year one residency seats in each field within the United States and Canada during the 2018 match year.

The student and rare volunteer interactions were analyzed to determine the type of interaction between pair individuals: in person versus telecommunication versus email. Statistical analysis incorporating the mean of each annual program iteration was included in an analysis of variance (ANOVA), with a post-hoc Holm-Sidak’s multiple comparison test. Rare volunteers were analyzed using simple qualitative metrics. All survey data were summarized using descriptive statistics.

## 3. Results

### 3.1. Program Uptake

**Figure 1:**
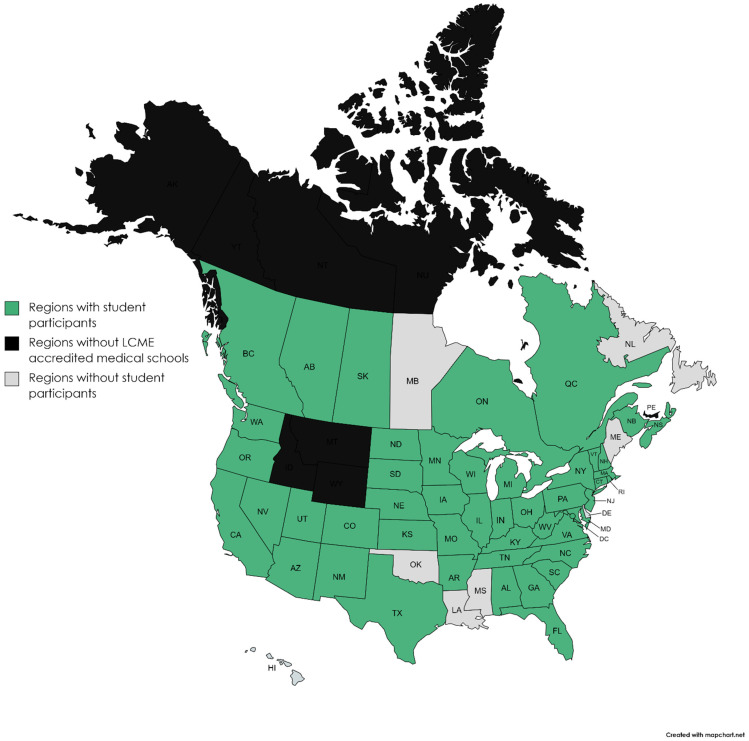
Map of United States and Canada, demonstrating regional uptake of the RARE Compassion Program between 2014 and 2018. States and provinces from which students participated in the program are indicated in green, while those without representative participants are indicated in grey. Notably, Alaska (AK), Wyoming (WY), Montana (MT), Idaho (ID), Prince Edward Island (PE), Yukon Territories (YT), Northwest Territories (NT) and Nunavut (NU) indicated in black do not have LCME accredited medical schools.

**Figure 2:**
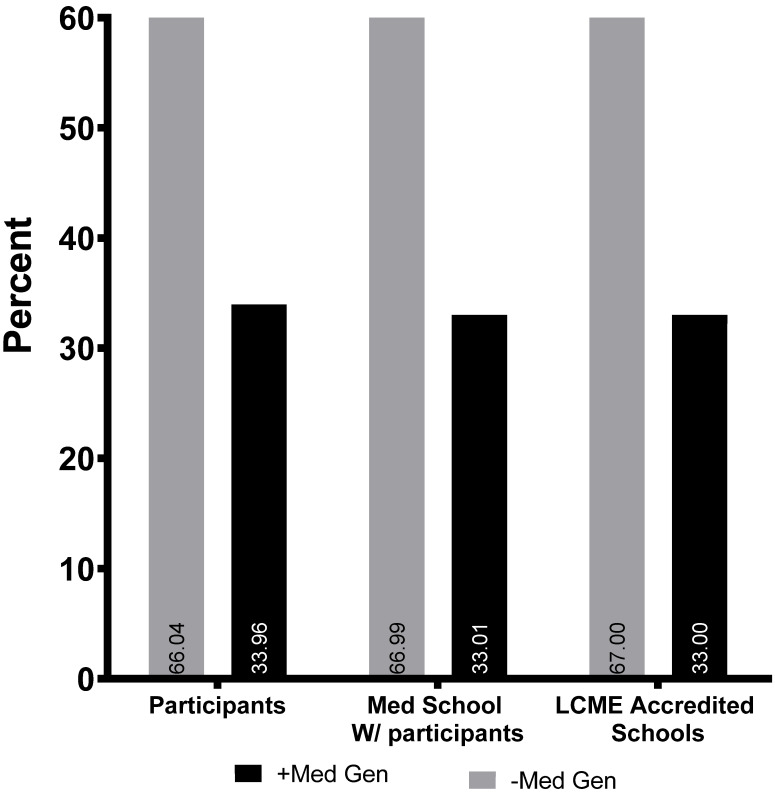
Percentage of student participants grouped by medical schools with and without associated post-graduate medical genetics training programs (which was used as a marker of rare disease centers/infrastructure), represented by black and gray bars respectively. To minimize the impact of variable medical school class sizes, program participation was also compared using individual medical school. The *x*-axis cohorts correspond to a *y*-axis of percent student participants and percent medical schools with participating students. Percentage of LCME accredited medical schools was used as a control on the far right.

Between 2014 and 2018, 334 students signed up for the RARE Compassion Program and were matched with rare volunteers. These 334 students represent uptake by 67.3% of AAMC member medical schools (Figure 1). 33.96% of participating students attended schools with affiliated rare disease centers. This is comparable with the proportion of eligible North American medical schools with associated rare disease centers (33.4%, Figure 2).

### 3.2. Program Volunteers and Interactions

**Figure 3:**
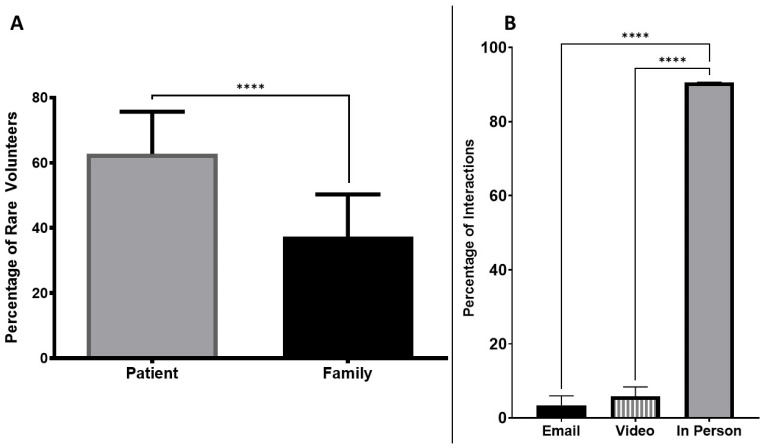
Characterization of student-rare volunteer interactions, with the Y axis representing the percentage of student participants who were pared with individual rare patients vs. family volunteers (Panel (**A**)), and the mechanism of interaction opted for (Panel (**B**)). **** denotes *p*-values of <0.05.

During the study period, there were 5389 rare volunteers who signed up to be matched with students, or 16.43 rare volunteers per student. The majority of volunteers were individual patients (62.7%), rather than patients and their families (37.3%) (*p* = 0.0337, Figure 3). 44.64% of rare volunteer self-identified as pediatric patients while and 53.36% identified as adults.

Despite not requiring in-person interaction, 90.64% of student-rare volunteer interactions were in-person, while only 5.89% and 3.46% were by video messaging or email correspondence (*p* = 0.0002, Figure 3).

### 3.3. Participant Career Selection

Of the 334 student participants, 112 students had residency match data available (33.5%). Nineteen out of 27 available residency fields were represented amongst the graduates, with primary care fields (Internal Medicine, Pediatrics, Obstetrics and Gynecology, and Family Medicine) being the most popular among participants (Table 1). Cardiovascular Surgery, Medical Microbiology, Nuclear Medicine, Pediatric Neurology, Ophthalmology, Physiatry, Public Health, Radiation Oncology, Diagnostic Radiology, and Vascular Surgery were underrepresented among matched participants based on the relative proportion of annual residency positions. Theses ten fields however only account for 4.5% of residency positions (Table 1).

### 3.4. Post-Participation Survey

A limited post-participation survey, with a 2.6% response rate (n = 12), was used in 2018 to determine student impressions of the RARE Compassion Program. Respondents had an overall positive experience from the scholarship, giving an average ranking of 4.27 out of 5 (95% CI 3.91–4.64). Further, 91.3% of respondents stated they would recommend the program to their peers. Notably, 50% of participants reported plans to advocate for rare disease education within medical schools, and 58.3% of participants indicated plans to become involved in rare disease advocacy after medical school.

## 4. Discussion

Individuals with rare diseases are a significant but often underappreciated segment of the population [6,8]. Multiple studies have highlighted the lack of direct exposure to rare disease patients, and need for enhanced medical education regarding rare diseases during medical training [6,7,19,21]. Given that rare patients often have a unique clinical experience relative to other chronic disorders, it is unsurprising that the AAMC highlighted the need for education in understanding and management of rare patients [5,10,14]. This paper provides a retrospective program analysis of The RARE Compassion Program, a voluntary education program which addresses this unmet growing need, and gives greater exposure to the unique patient experience associated with rare diseases.

Despite being administered by a third party without a direct channel through which to communicate with most eligible medical students, the program has thus far demonstrated widespread uptake among medical schools (Figure 1 and Figure 2). While most of these academic centers have individual rare disease specialists, only 33.4% of schools have established postgraduate medical education programs in medical genetics, which we used as a marker of a rare disease center. Notably, uptake at this handful of medical schools was not overrepresented, despite greater potential exposure to, and investment in, rare diseases (Figure 2).

Peer-to-peer endorsement may have contributed to the fast uptake of the program across the majority of medical schools in Canada and the United States. The limited survey data included in this program evaluation revealed an overwhelming 91.7% of students indicated that they would recommend the program to their peers. The strong positive rating of 4.27/5 could relate to the direct contact with patients and/or their families, which would enhance the didactic teaching more typical of rare disease education [22,23].

It is well established that patient interactions can be transformative for a trainee still early in their career, and leader to patient-centered providers [23,24,25,26,27,28,29,30,31]. The RARE Compassion Program was designed to stimulate future interest in rare disease patient care and advocacy from within the medical community. Encouragingly, this limited survey showed that the majority of respondents developed an interest in including rare disease advocacy in their future career. While multi-year follow-up would support these findings, this work suggests that the Rare Compassion Program has thus far been successful in stimulating rare disease interest amongst future medical providers.

Importantly, with unrepresented specialties accounting for only 4.5% of residency positions, the scholarship demonstrated its appeal to students pursuing nearly every field of medicine. Nuclear Medicine, Medical Microbiology, and Public Health Medicine account for 3 of the 8 unrepresented residency programs; however, their programs are only direct entry in Canada and thus not part of the NRMP [20]. Given that the care of rare patients often includes a highly diversified team of specialists, it is particularly important that the program, and rare disease education more broadly, not be limited to the primary care and genetic fields [5,6,9,10,16,32].

Students with an interest in primary care fields (Family Medicine, Internal Medicine, Pediatrics, Obstetrics and Gynecology) were best represented (Table 1). However, Family Medicine was underrepresented among participants when considering overall number of residency seats (representing 8.9% of participants compared to the allocated 15.6% seats). This could be related to the relative competitiveness of different specialties, which likely also influenced representation of certain fields more than others [20,33,34]. The majority of fields that were overrepresented relative to their residency seats, for example, are traditionally considered to be highly competitive (Dermatology, Neurological Surgery, Otolaryngology, and Plastic Surgery) [20,33,34]. The higher than expected participation of students entering these competitive specialties could be due to the hope that engaging in this kind of program might bolster one’s residency application. Alternatively, it may be that the personality type drawn to applying for scholarships and programs of this kind may also be drawn to fields perceived as ambitious or competitive. Importantly, several fields traditionally associated with diagnosing and caring for rare patients were also well represented, such as Obstetrics, Pediatrics, Internal Medicine, and Pathology [6,35]. As the preliminary survey did not solicit the motivations behind participation, further work is necessary to properly address the question of specialty choice and its influences.

This study is limited by the self-selected nature of opt-in participation, in both the program itself and the survey. This may have introduced a bias to respond favorably to questions around the importance of rare disease education, as well as to the pursuit of careers relating to rare disease. Further, there was no pre-participation survey for this cohort, so it is challenging to assess the impact of the program on participants. The study also lacks long-term follow-up data. For additional program evaluation and quality improvement, Global Genes has instituted an optional pre- and post-participation survey for student participants in the RARE Compassion Program. Therefore, future data will provide greater insight into motivation and the multifaceted impact of participation on these students. Although this program, and much of the literature that informs the study, is limited to the North American context, the results remain generalizable to global medical education community. Although the nature of medical education differs across jurisdictions, this program highlights the positive impact of an extra-curricular experiential education program to complement student learning about rare diseases. This is particularly valuable where current medical school curricula worldwide have limited space for new topics, despite rapid expansion in our knowledge of rare diseases and the need to teach it to students.

## 5. Conclusions

This retrospective program evaluation demonstrates that the RARE Compassion Program and the David R. Cox Scholarship, designed to increase medical student exposure and understanding around patients with rare disease, have broad appeal. This serves as a novel case study of how extracurricular initiatives supported by non-profit organizations can augment the medical training experience and improve learning around important topics, such as care for patients with rare diseases. Furthermore, this study demonstrates that students destined for both primary and specialty care were well represented among participants. Further research into the pre-existing motivations and subsequent program influence on career trajectory will be essential to understanding the true impact of this program on the rare disease community and its experience of the healthcare system.

## Figures and Tables

**Table 1:** Percentages of participants per field of study, compared to percentage of residency positions in that field. Seats per United States Medical Doctorate graduate (USMD) applicant, as reported by NRMP, was used as a marker of program competitiveness. Fewer seats per USMD correlate with more competitive fields.

Field	Percentage of Participants	Percentage of Residency Positions	Seats per USMD Applicant Where Reported from the NRMP [20]
Internal Medicine	19.64	25.98	2.4
Pediatrics	17.86	9.27	1.7
Obstetrics and Gynecology	9.82	4.39	1.1
Family Medicine	8.93	15.61	3.1
Emergency Medicine	5.36	7.29	1.4
General Surgery	5.36	4.34	1.3
Anesthesiology	5.36	6.05	1.4
Neurology	4.46	2.78	2
Psychiatry	4.46	5.39	1.5
Pathology	3.57	2.03	2.9
Combined Internal medicine pediatrics	2.67	1.19	1.1
Dermatology	2.67	1.55	1.2
Otolaryngology	2.67	1.06	0.8
Neurological Surgery	1.79	0.75	0.9
Orthopedic Surgery	1.79	2.45	1.0
Radiology	0.89	3.65	1.6
Direct Entry Medical Genetics	0.89	0.02	Not part of the NRMP
Plastic Surgery	0.89	0.58	0.8
Urology	0.89	1.09	Not part of the NRMP
Cardiothoracic Surgery	0	0.03	0.7
Medical Microbiology	0	0.02	Not part of the NRMP
Nuclear Medicine	0	0.02	Not part of the NRMP
Ophthalmology	0	1.59	Not part of the NRMP
Physiatry	0	1.39	1.8
Public Health	0	0.02	Not part of the NRMP
Radiation Oncology	0	0.65	1.6
Vascular Surgery	0	0.21	1
